# Changes in Environmental Conditions Differentially Affect the Bacterial Microbiome Communities in Different Apple Fruit Tissues

**DOI:** 10.1111/1758-2229.70225

**Published:** 2025-11-27

**Authors:** Michael S. Mclaughlin, Svetlana N. Yurgel, Pervaiz A. Abbasi, Shawkat Ali

**Affiliations:** ^1^ Department of Plant, Food, and Environmental Sciences Faculty of Agriculture, Dalhousie University Truro Nova Scotia Canada; ^2^ Kentville Research and Development Centre Agriculture and Agri‐Food Canada Kentville Nova Scotia Canada; ^3^ United States Department of Agriculture (USDA), Agricultural Research Service, Grain Legume Genetics and Physiology Research Unit Prosser Washington USA

**Keywords:** apple, bacterial microbiome, environmental factors, management practices, microbial network

## Abstract

The maintenance and manipulation of the beneficial plant microbiome is a new frontier in ecofriendly disease management, particularly during post‐harvest storage. However, the fruit microbiome is highly variable and can be influenced by both biotic and abiotic factors. A comprehensive understanding of how these factors influence microbial communities is necessary in order to unlock the microbiome for sustainable disease management. In this study, we demonstrate the impacts of the growing season and management strategy on the composition and structure of the bacterial microbiome of ‘Honeycrisp’ apples at harvest from seven different orchards in the Atlantic Maritime Ecozone, over the course of two growing seasons. We show that the bacterial communities associated with core and peel tissues respond differently to changes in external environmental conditions, underscoring the need to include multiple tissue types in future fruit microbiome research. Finally, we characterize the microbial cooperation networks of apple core and peel tissues and identify key microbial taxa influencing these networks.

## Graphical abstract

This study investigates the bacterial communities in ‘Honeycrisp’ apples in Atlantic Canada, highlighting how weather, and farming practices shape these communities in different fruit tissues. Weather impacts core and peel tissues bacteria differently across growing seasons, identifies key microbes in microbial interaction networks, and provides insights into using these communities for disease control.

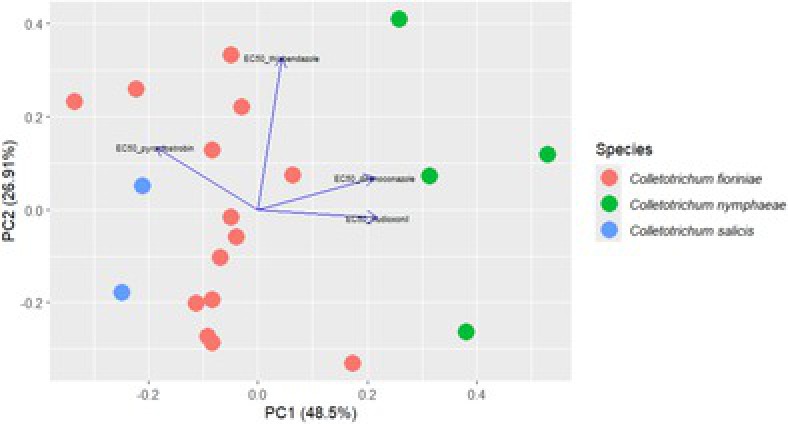

## 1 Introduction

The domesticated apple ( *Malus domestica* ) is a historically, culturally, and economically significant food crop grown in temperate areas globally (Spengler 2019). In the past decade, apple production has grown immensely from approximately 83.1 million metric tons in 2017 to approximately 95.84 million metric tons in 2022 (Shahbandeh 2022). In 2019, the global apple market reached a value of US$78.8 billion (Indexbox 2022). Apple is a particularly important crop for the Canadian agricultural industry, with a farm gate value of $259.1 million in 2020 (Agriculture and Canada 2021). Apple fruit is vulnerable to pre‐ and post‐harvest fungal pathogens which can significantly reduce the yield of fruit and/or render the fruit unmarketable. For instance, apple scab, caused by the fungal pathogen *Venturia Inaequalis (Cke.)* Wint, can cause severe deformities of apple fruit, as well as early fruit fall, resulting in losses of up to 70% (Biggs et al. 1990; MacHardy 1996). In the post‐harvest stage, the majority of all fruit losses are caused by phytopathogenic fungi. In the United States, it is estimated that losses from post‐harvest diseases are close to 20% every year (Gupta and Saxena 2023). Post‐harvest losses from fungal diseases may reach 50% in developing countries that lack effective management programmes (Dutot et al. 2013). For decades, the primary means of suppressing fungal pathogens of apple fruit has been the use of chemical fungicides. However, concerns over the environmental and human health impacts have led to increased scrutiny, reducing the number of applications per crop of fungicides and increasing the re‐entry interval time (Ragsdale and Sisler 1994; Steinhauer et al. 2018). Therefore, more eco‐friendly alternative means of disease management are clearly necessary.

It is well established that plant‐associated microbiota exert significant influences on the physiological traits of their host and can negatively or positively influence plant health, development, and resistance to biotic and abiotic stresses (Berg et al. 2015). These microbial communities have evolved in close association with their host plant and are partially influenced by its genetics (Rosenberg and Zilber‐Rosenberg 2016; Lyu et al. 2021). Mutualistic plant‐associated microbiota can directly or indirectly antagonize pathogens either by competition for physical space and resources within the host (Gunatilaka 2006) and/or by secretion of antimicrobial compounds (Raaijmakers and Mazzola 2012), and/or by inducing the host's defence mechanisms (Redman et al. 1999), and by mycoparasitism of pathogenic microbiota (Huang et al. 2020). As such, the selection of promising plant‐associated microbiota for use as biological control agents has become an attractive alternative in order to reduce the use of conventional fungicides (Quimby et al. 2002; Griffin 2014). As only a small number of genera have currently been identified as suitable for use in effectively managing plant disease, the continued identification of potential biocontrol agents is a key goal in the advancement of effective biocontrol.

Beyond the contributions of individual microbiota, there is additional evidence that the diversity and structure of plant‐associated microbial communities can influence plant health, and that disruptions in these communities can be correlated with disease. For instance, Fusarium wilt disease of tomato was shown to be negatively correlated with the diversity of bacterial and fungal communities in the rhizosphere, while a loss in diversity in the microbial communities of mango fruit was associated with increased susceptibility to stem‐end rot caused by *Alternaria alternata* (Diskin et al. 2017; Zhou et al. 2022). Thus, the diversity and structure of the fruit microbiome may serve as potential indicators of fungal disease susceptibility. However, analyses based solely on diversity and community structure offer limited resolution and may fail to capture the complex dynamics of microbial ecosystems. The stability and population dynamics of microbial communities can be heavily influenced by interactions among microbial community members, which can be either positive, negative, or neutral. As such even minor shifts in the relative abundance of an individual microbe can significantly influence the broader microbial community (Czárán et al. 2002; Kinkel et al. 2011; Friedman et al. 2017; Banerjee et al. 2018). Therefore, the exploration of microbial co‐operation and interaction networks has emerged as a valuable approach for evaluating the resilience of microbial communities (Karimi et al. 2017; Schlatter et al. 2018; Esan et al. 2019).

It has been suggested that disease control could be achieved through maintaining the microbiome of the host plant within a “healthy” state (Adam et al. 2016; Berg et al. 2020). In order to achieve this goal, a thorough understanding of microbial community dynamics is important. This is particularly difficult, as numerous biotic and abiotic factors can influence plant‐associated microbial communities, particularly in the phyllosphere, which is more exposed to changes in the external environmental conditions (Bera et al. 2024). In the case of apple fruit, numerous factors such as growing season, management practice, geographical location, cultivar, pre‐storage treatments and storage duration have been shown to influence the structure and diversity of fruit‐associated microbial communities (Abdelfattah et al. 2016, 2021; Wassermann, Muller et al. 2019; Bosch et al. 2021; Britt et al. 2021). In many of these studies, these variables were examined in isolation, necessitating a more comprehensive investigation of their relative impacts on the microbial communities of apple fruit. In addition, many earlier studies on the fruit microbiome have relied on a single tissue type, though it has now been demonstrated that differences in the tissue micro‐environment significantly influence microbial communities; therefore, the communities of one tissue type may not be representative of those of the fruit as a whole, necessitating the inclusion of multiple tissue types in future research (Wassermann, Muller et al. 2019; Abdelfattah et al. 2021). Thus, in order to develop microbiome‐based strategies for disease control, a comprehensive understanding of how common abiotic factors influence the stability, structure, and function of the microbial communities of different tissue micro‐environments will be necessary.

In our previous study, we have demonstrated that environmental conditions between growing seasons seemed to have the greatest impact on the fungal communities of ‘Honeycrisp’ apples when compared to plant tissue type, management practices, and geographical location (McLaughlin et al. 2023). However, existing literature suggests that fungal and bacterial communities may not respond in the same manner to these factors. Notably, bacterial communities tend to display higher stability relative to the fungal communities following different pre‐storage treatment regimens, and show reduced variability between geographical locations (Wassermann, Kusstatscher et al. 2019; Abdelfattah et al. 2020, 2021). In this study, we characterized the bacterial microbiomes of the core and peel tissue of ‘Honeycrisp’ ( *M. domestica* ) apples across the Atlantic Maritime Ecozone. We compared the relative impacts of tissue type (core and peel), management practices (organic and conventional) and changes in environmental conditions between growing seasons (2019, 2020) on the structure and composition of bacterial communities of apples. In addition, we define the CORE bacterial microbiota that are consistently present in ‘Honeycrisp’ fruit and explore microbial co‐occurrence and co‐exclusion within fruit tissue, identifying the key taxa in these interactions. Finally, we compare the predicted functional pathways associated with microbial communities in different fruit tissues, highlighting notable differences in their potential metabolic capacities.

## 2 Experimental Procedures

### 2.1 Site Description and Agronomic Management Information

The experiment utilized commercial orchards across Maritime Canada. The orchard sites used in this experiment, including their geographical locations and management practices, have been described previously (McLaughlin et al. 2023). In conventional orchards, 12–14 sprays of synthetic fungicides were applied over the course of the growing season, while the organic orchards were managed according to approved organic practices.

### 2.2 Plant Material and Sample Preparation

The DNA used in this experiment was prepared in a previous manuscript. Briefly, the experiment was conducted with ‘Honeycrisp’ fruits from five conventional orchards in Maritime Canada—two in PEI, two in Nova Scotia, and one in New Brunswick, as well as two organic orchards in Nova Scotia. The fruit were collected at commercial maturity in late September and early October 2019 and 2020, respectively. A comprehensive list of the samples used in the dataset can be seen in Table S1. At each sampling time, five fruit were collected from eight non‐adjacent healthy apple trees per orchard, and the fruits of each tree were combined to form a single sample. Core samples consisted of 3 cm of both the cores from stem and calyx‐ ends, which were removed from the apple with an apple corer, while peel samples consisted of 3 cm of peel from around the circumference. Both core and peel samples were immediately flash frozen in liquid nitrogen. Thus, the calyx and stem ends of five fruit, and the apple peels from said fruit, were combined into two separate tissue‐type samples (core and peel samples), making eight biological replicates of each tissue type per orchard per growing season. The core and peel samples were then freeze‐dried using a lyophilizer at −80°C for 72 or 24 h, respectively. Following grinding in liquid nitrogen with a mortar and pestle, approximately 100 mg of lyophilized tissue was used for DNA isolation using the QIAGEN Power Soil DNA extraction kit (Cat No. 12888‐100) according to the manufacturer's protocols.

### 2.3 PCR Amplification of Target DNA and Illumina Sequencing

The V4 region of the bacterial 16S region was amplified using the 16S primers 515FB (5′‐GTGYCAGCMGCCGCGGTAA‐3′) and 806RB (5′‐GGACTACNVGGGTWTCTAAT‐3′) (Walters et al. 2016). In order to prevent the amplification of mitochondrial and ribosomal sequences, these primers were used in conjunction with Peptide Nucleic Acids (PNAs) (PNA Bio) (Lundberg et al. 2013). PCR reactions consisted of 0.25 μL of *Pfu* polymerase (4 units/μL), 1 μL of each 515F forward primer (10 μM), 806RB reverse primer (10 μM) and 2.5 μL each of plastid and mitochondrial PNAs (5 μM). In addition to this, 2.5 μL of template DNA, 2.5 μL of 10× *Pfu* buffer and 12.75 μL of nuclease‐free water were added per reaction for a total reaction volume of 25 μL. Reactions were incubated in a C1000 touch thermocycler for 95°C for 5 min, followed by 40 cycles at 95°C for 30 s, 78°C for 5 s, 55°C for 30 s, 72°C for 1 min and a final extension of 72°C for 8 min. To confirm successful amplification, the completed reaction was subsequently run on a 0.5% agarose gel, and the ~350‐bp band was purified using a QIAGEN gel extraction kit (QIAEX II Gel Extraction Kit Cat No. 20051) following the manufacturer's protocol, and 10 μL of each purified reaction was sent for Illumina Sequencing to Dalhousie University's CGEB‐IMR lab (https://www.imr.bio). The reference primers 515FB and 806RB were used to amplify the V4 region of the bacterial 16S region. An Illumina Miseq platform was used to multiplex the sequences through dual‐indexing in order to generate 2× 300 bp paired‐end reads as described in Comeau et al. (2017). The raw sequencing data are available at https://www.ncbi.nlm.nih.gov/sra/PRJNA1213154.

### 2.4 Bioinformatics and Statistical Analysis

Initially, raw amplicon‐sequencing reads were inspected using FASTQC (v0.11.4). The primer sequences were trimmed using the Qiime2 plugin ‘cutadapt’ version 2023.8 (Martin 2011). Following primer trimming, analyses were carried out within QIIME 2 version 2023.8 (Bolyen et al. 2019), using Qiime2‐Deblur in order to trim reads to a length of 253 bp. ASVs were classified taxonomically using a Naïve‐Bayes RDP classifier and accessing the SILVA 16S database v138 (Quast et al. 2013). ASVs with a mean relative abundance of less than 0.1% of the dataset were removed in order to account for the internal sequencing error for the Illumina MiSeq platform, resulting in the exclusion of ASVs below 26 reads, following which plant‐derived ASVs (e.g., chloroplasts and mitochondria) were also removed. Depending on the variable, different subsets of data were examined as previously described (McLaughlin et al. 2023).

QIIME2 version 2023.8 was used to calculate core taxa (present in 75% of all samples or more), relative abundances, as well as alpha and beta‐diversity indices. To determine the degree of variance that could be explained by a given variable (tissue type, management regime, location, growing seasons), ADONIS tests were conducted within QIIME2 using a weighted Unifrac Distance Matrix. To view differences in bacterial community structure, non‐multidimensional scaling analyses of bacterial communities based on ASVs were conducted in R (version 4.2.3). Likewise, ALDEx2 1.24.0 was used to identify differentially represented features within R (Fernandes et al. 2013). Significant results were based on a Benjamini‐Hochberg multiple test correction of a Welch's *t*‐test (Tissue, Treatment, Year) or Kruskal–Wallis test (Geographical Location).

In order to prepare ecological networks, inverse covariance was calculated using the Sparse Inverse Covariance Estimation for Ecological Association Inference (SpiecEasi) R package (Kurtz et al. 2015). The bacterial data from this manuscript were combined with the fungal dataset for matching samples previously published in McLaughlin et al. (2023). In this respect, the core and peel samples were divided into two separate networks, and a total of 80 samples with sufficient depth of both bacterial and fungal reads were selected for both networks. Interactions with *p* values exceeding 0.05 and with weights below 0.1 were excluded from the dataset, and the networks were visualized using Cytoscape (Shannon et al. 2003). In both networks, individual taxa are represented as nodes, and the interactions between taxa are represented as positive (red) and negative (blue) edges. Both networks were visualized as “edge‐weighted spring‐embedded” co‐occurrence networks.

The functional potential of bacterial communities was predicted using the software PICRUSt2 (Douglas et al. 2020). The estimated relative abundances of predicted pathways were rounded and differentially represented pathways were calculated using Aldex2 as described above (Fernandes et al. 2013). Statistically significant results were based on a Benjamini‐Hochberg multiple test correction of a Welch's *t*‐test.

## 3 Results

### 3.1 The Overall Bacterial Diversity and Compositional Structure of Apple Fruit

In total, 2,891,125 16S rRNA reads were obtained following the removal of host sequences as well as amplicon sequence variants (ASVs) with a mean abundance of less than 0.1%. These sequences comprised 769 unique ASVs, representing 15 bacterial phyla, 22 classes, 55 orders, 111 families, and 212 bacterial genera. Proteobacteria (82% of total reads (TR)), Actinobacteria (11% of TR) and Bacteroidota (6% of TR) dominated the bacterial phyla, while Gammaproteobacteria (50% of TR), Alphaproteobacteria (32% of TR) and Actinobacteria (11% of TR) were the most abundant classes. Pseudomonadales (30% of TR) was the most abundant order, followed by Rhizobiales (15% of TR) and Enterobacterales (126% of TR), and correspondingly the most relatively abundant families were the Pseudomonadaceae (29% of TR), Sphingomonadaceae (16% of TR) and Rhizobiaceae (16% of TR). At the genus level, *Pseudomonas* (29% of TR), *Allorhizobium–Neorhizobium–Pararhizobium–Rhizobium* (12% of TR) and *Sphingomonas* (10% of TR) were dominant. A complete breakdown of the resolved taxa from the dataset is provided in Table S1.

### 3.2 Composition of the Bacterial Communities in Core and Peel Tissues

In order to determine differences in bacterial community structure between core and peel tissue of ‘Honeycrisp’ apples, we selected 30 peel samples with the highest sequencing depth, and the 30 corresponding core tissue samples, rarefying to a depth of 5335 reads for further analysis. This was necessary as the core and peel samples had immense differences in their sequencing depths so rarefying at a low number of reads, based on peel samples, would result in a loss of many unique ASVs within the core tissues, and thus heavily influence the data analysis.

**FIGURE 1:**
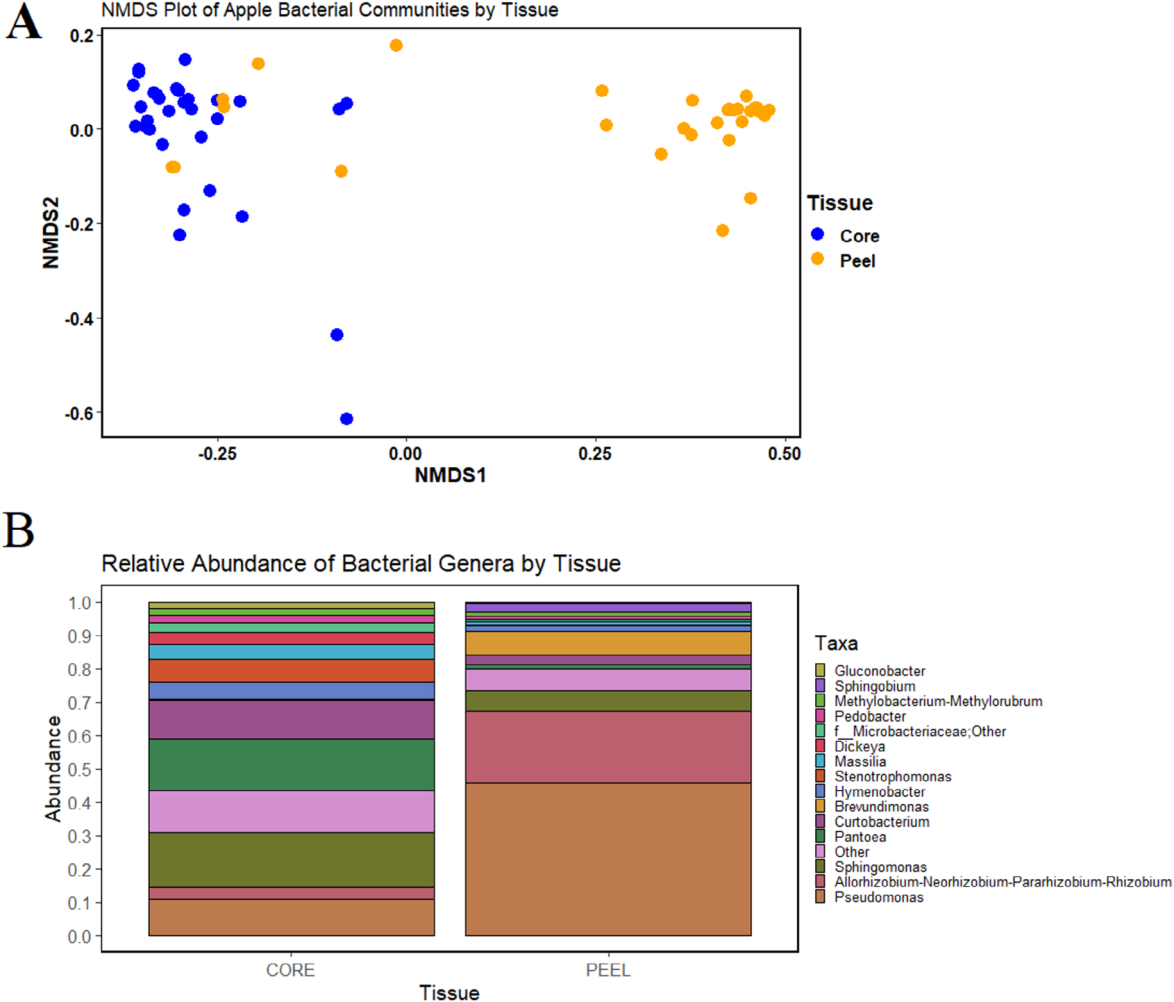
The impact of tissue type on the bacterial communities of apple. (A) NMDS analysis of the bacterial communities of core and peel tissues based on individual ASVs. (B) The relative abundance of the top 15 most abundant bacteria by tissue type.

We analyzed the impact of tissue type on microbial community composition. This factor accounted for around 32% of the difference between core and peel communities (*R* ^2^ = 0.317, *p* < 0.001). These samples formed unique clusters based on tissue type within a Non‐Metric Multi‐Dimensional Scaling (NMDS) analysis, with minor overlap (Figure 1A). Significant differences were observed in alpha diversity indices between tissue types, as peel tissues were characterized by a lower Shannon diversity, evenness, and a lower number of total ASVs (Table 1).

**TABLE 1:** Alpha‐diversity of bacterial community.

Tissue type	Evenness	Shannon‐diversity	Observed features
Core	0.59A	4.28A	151.94A
Peel	0.47B	3.21B	117.16B
Cores			
Management strategy			
Organic core	0.55A	3.63A	97.03A
Conventional core	0.49B	3.11B	78.04B
Growing season			
2019	0.58A	3.92A	110.8A
2020	0.55A	3.73A	115.41A
Peels			
Management strategy			
Organic peel	0.62A	3.99A	84.66A
Conventional peel	0.71B	4.8B	111.08B
Growing season			
2019	0.7A	4.78A	115.08A
2020	0.6B	3.72B	76.13B

*Note:* Letters denote statistically groupings based on Student's *t*‐test. Different letters denote statistically significant differences (*p* < 0.05).

In both tissue types, the top 15 most relatively abundant genera accounted for the majority of TR, namely 85% and 90% of TR in core and peel tissues, respectively (Figure 1B). However, the most abundant genera differed between tissue types. For example, *Pseudomonas* and *Allorhizobium–Neorhizobium–Parathizobium–Rhizobium* accounted for 46% and 22% of the TR in peel tissues but only 11% and 3% of the TR in core tissues. *Sphingomonas* was the most relatively abundant genus in core tissues, accounting for 17% of the TR in comparison to only 6% of the TR in peel tissues. Fifty‐one differentially represented bacterial genera were observed (we.eBH *p* < 0.05, Table S2), which included nearly all of the top 15 genera, excluding only *Methylobacterium–Methylorubrum*.

### 3.3 Orchard Management Practices Influenced Fruit Bacterial Communities

In order to examine the bacterial communities of core and peel tissues individually and preserve unique ASVs within core tissues for downstream analysis, we separated the 112 corresponding core and peel samples into separate datasets. Core samples were rarefied to a sampling depth of 4044 reads, while peel samples were rarefied to a depth of 1138 reads. These two datasets were then used to examine the influence of management practices, and the environmental conditions of different growing seasons and geographical locations on bacterial communities of core and peel tissues, respectively.

**FIGURE 2:**
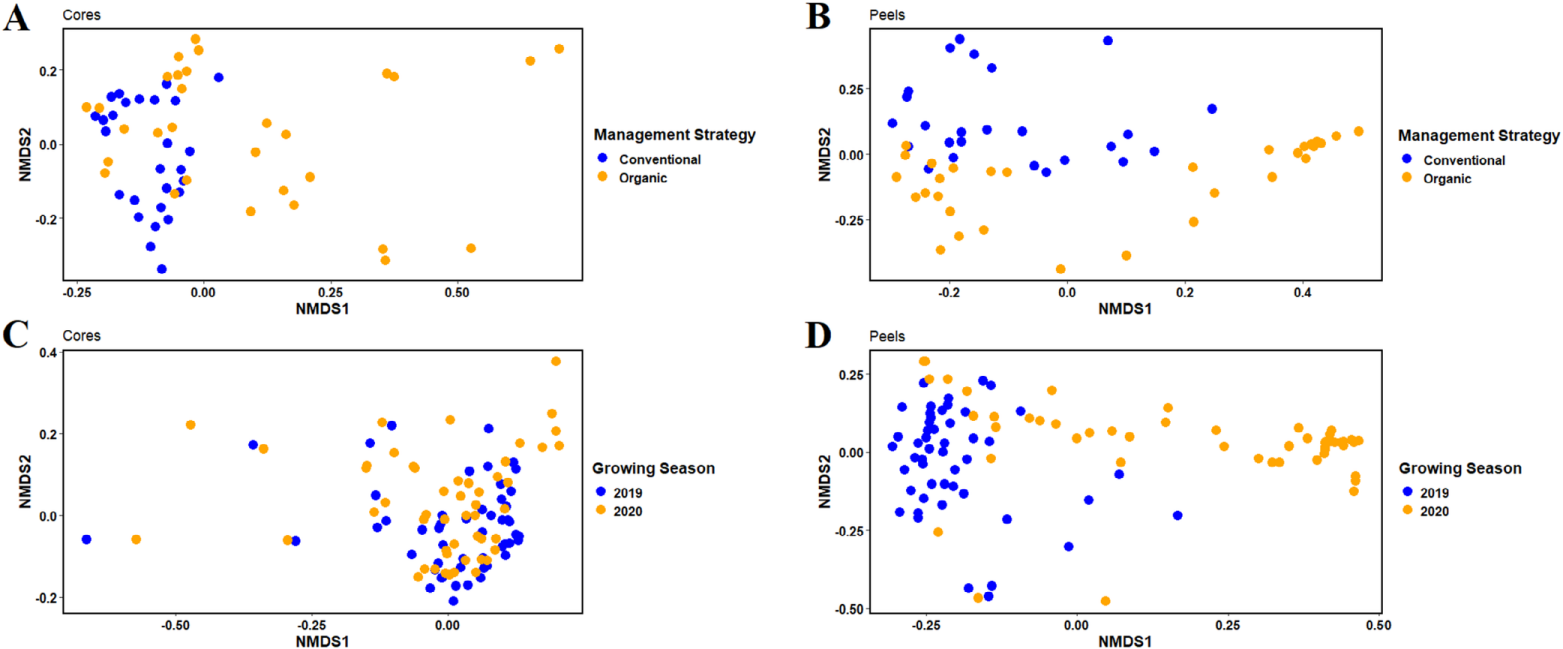
Management practices and growing season differentially affect community composition across different tissues. NMDS analysis of the bacterial communities of apples based on individual ASVs and clustered by organically and conventionally managed core (A) and peel tissues (B), and by growing season, with core (C) and peel (D) tissues harvested during the 2019 and 2020 growing seasons, respectively.

To determine the impact of management practices on the bacterial communities of ‘Honeycrisp’, we compared the bacterial communities of apple fruits from two organically and two conventionally managed orchards in Nova Scotia. An analysis of the strength and significance of sample groupings indicated that management practices had a significant impact on community composition (*R* ^2^ = 0.100, *p* < 0.01), while an NMDS analysis demonstrated that the communities of organically managed fruit were more dispersed than the communities of conventionally managed fruit (Figure 2A). Management‐driven differences were also apparent in the alpha‐diversity indices, with organically managed core communities being characterized by higher Shannon diversities, evenness, and number of observed features compared with conventionally managed core communities (Table 1).

**FIGURE 3:**
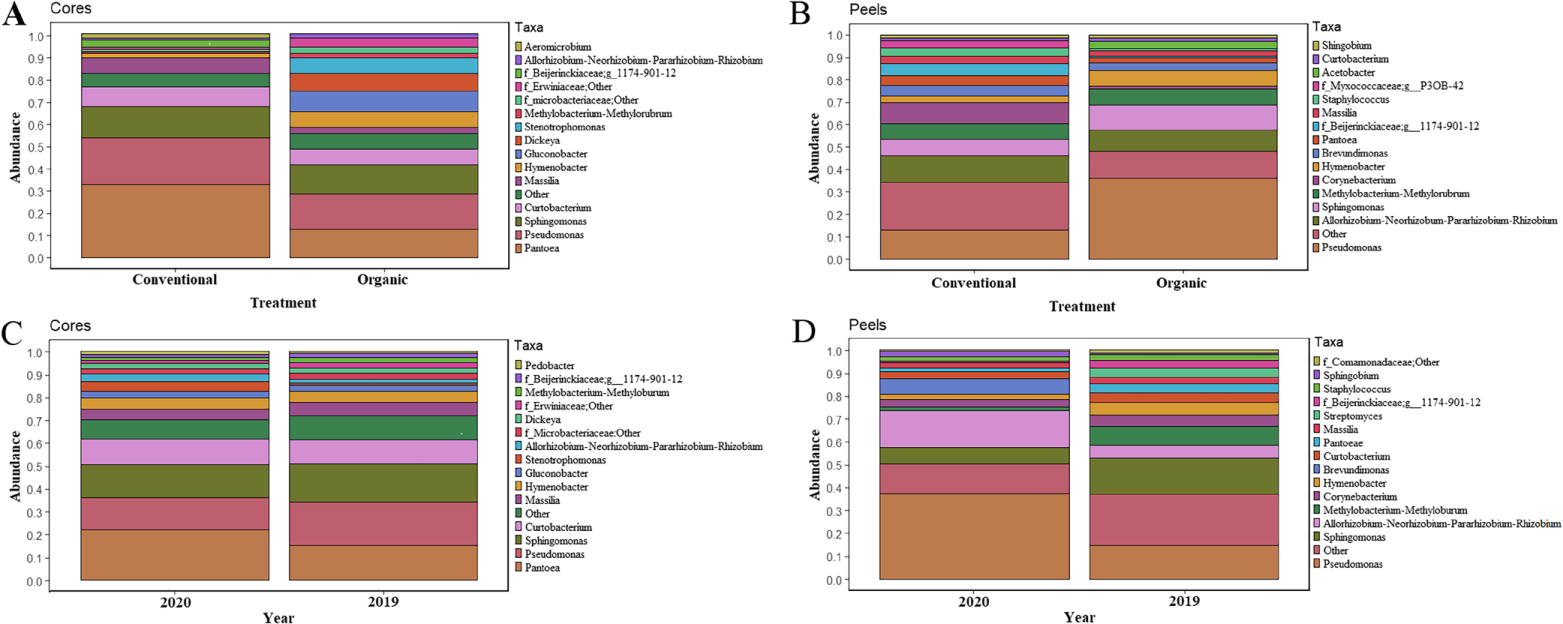
Management practices and growing season influence the relative abundance of bacterial microbiome. Relative abundance of the top 15 most abundant bacterial genera in apple fruit. The samples were grouped by conventionally and organically managed core (A) and peel (B) tissues, and by growing season, with core (C) and peel (D) tissues harvested during the growing seasons of 2019 and 2020, respectively.

The top 15 genera were represented by more than 95% of the TR in both conventionally and organically managed core communities (Figure 3A). For both communities, *Pantoea* and *Pseudomonas* were the most abundant genera. However, the relative abundance of these two generally differed significantly between treatment types. In conventionally managed tissues, these genera accounted for 33% and 21% of the TR compared with 13% and 16% of the TR in organically managed core tissues, respectively. *Pantoea* was the most relatively abundant genus in conventionally managed core tissues, while *Pseudomonas* was the most relatively abundant genus in organically managed core tissues. Twelve differentially represented genera were identified (we.eBH *p* < 0.05, Table S3A) between organically and conventionally managed core tissues, including 7 of the top 15 genera.

Management practices also had a significant influence on the community composition of peel tissues (*R* ^2^ = 0.110, *p* < 0.001). The communities of organically managed peel tissues were more dispersed when observed by NMDS (Figure 2B). In contrast to core tissues, the conventionally managed peel tissues exhibited greater diversity than their organically managed counterparts, with significantly higher Shannon diversity, evenness and observed features (Table 1). While in both organically and conventionally managed peel tissues, a small number of genera dominated the TR. The top 15 genera accounted for 79% of the TR in conventionally managed peels, and 88% of the TR in organically managed peels (Figure 3B). Overall, *Pseudomonas* was the most relatively abundant genus, accounting for 36% of the TR in organically managed peel samples compared with 13% of the TR in conventionally managed peel samples. The next most relatively abundant genus was *Allorhizobium–Neorhizobium–Parathizobium–Rhizobium*, accounting for 12% and 10% of the TR in conventionally and organically managed peel tissues, respectively. Twelve differentially represented genera were identified (we.eBH *p* < 0.05, Table S3B) between organically and conventionally managed peel tissues, including 8 of the top 15 genera.

### 3.4 The Impact of Growing Season on ‘Honeycrisp’ Bacterial Communities

To investigate the influence of varying environmental conditions on the bacterial communities of ‘Honeycrisp’ fruit, we collected samples from the same locations in the 2019 and 2020 growing seasons. Compared with 2020, precipitation was significantly higher in 2019 (102 mm per month compared with 59 mm per month in 2020, *p* < 0.05). Intriguingly, this difference in precipitation was not reflected in changes to the bacterial community structure of core tissues (*R* ^2^ = 0.010, *p* < 0.2), and core tissue communities of both 2019 and 2020 seemed to cluster together when visualized by NMDS (Figure 2C). No differences in alpha‐diversity indices were observed in core tissue communities between growing seasons (Table 1). In both 2019 and 2020, the top 15 relatively abundant genera accounted for over 95% of the TR (Figure 3C), and only two differentially represented genera were identified between growing seasons (we.eBH *p* < 0.05, Table S4A), neither of which was represented in the top 15 genera.

In contrast to core tissue communities, the bacterial communities of peel tissues were significantly influenced by the growing season. A significant impact of the growing season was observed in the bacterial community structure of peel tissues (*R* ^2^ = 0.140, *p <* 0.001). Clear clusters can be identified for communities from 2019 and 2020, respectively, although the communities of peels harvested in 2020 appear to be more dispersed (Figure 2D). The growing season also significantly influenced the alpha diversity indices of peel tissue communities, with samples from 2020 exhibiting lower Shannon diversity, evenness, as well as a lower number of observed features (Table 1).

The top 15 most relatively abundant genera in peel tissues accounted for approximately 90% and 75% of the TR in 2020 and 2019, respectively (Figure 3D). Overall, 21 differentially represented genera were detected between peel tissue communities in the 2019 and 2020 growing seasons (we.eBH *p* < 0.05), six of which are among the top 15 most relatively abundant genera (Table S4B).

### 3.5 Network Analysis

**FIGURE 4:**
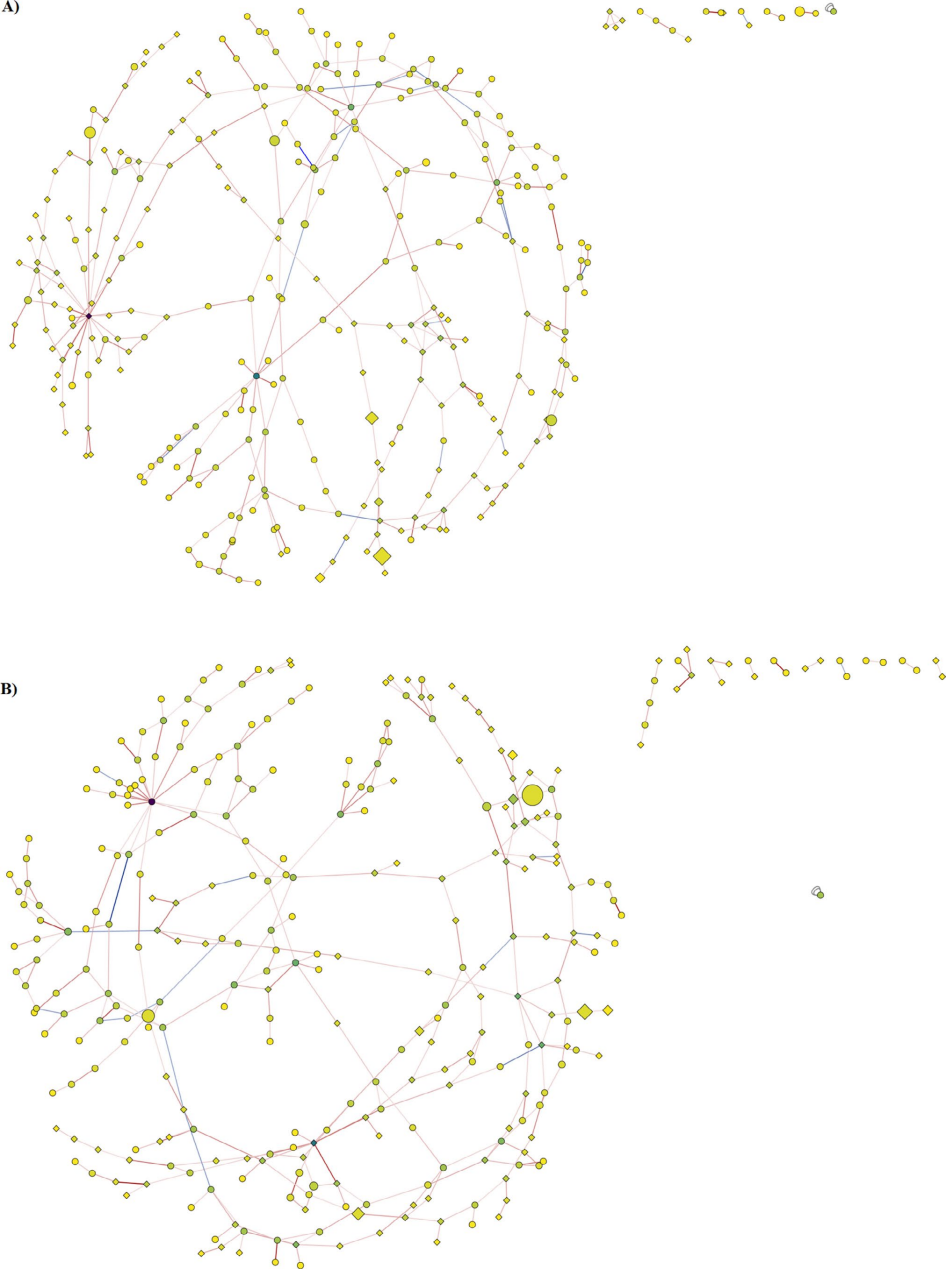
Inverse covariance networks showing potential interactions between fungal (diamond) and bacterial (oval) genera in ‘Honeycrisp’ core (A) and peel (B) tissues. The lines (edges) connecting the taxa represent positive (red) or negative (blue) co‐occurrence relationships, and the intensity of the colour represents the strength of the correlation.

Co‐occurrence and co‐exclusion networks for the communities of core and peel tissues were constructed (Figure 4A,B). In order to evaluate interkingdom (bacteria–fungi) interaction, we utilized data from our previous publication (McLaughlin et al. 2023) which examined the fungal apple fruit microbiome, of 80 samples of both core and peel tissues. The greatest sequencing depth represented in both fungal and bacterial datasets was used in the analysis. Within the core community network, interactions between bacteria made up the greatest proportion (47% of all interactions), followed by interactions between fungi (36% of all interactions) and interkingdom interactions (17% of all interactions). Thus, intrakingdom (bacteria–bacteria or fungi–fungi) interactions accounted for 83% of all interactions in the core community network. In the peel community network, interactions between fungi accounted for 25% of interactions, with interactions between bacteria and intrakingdom interactions accounting for 45% and 30% of all interactions, respectively.

Both core and peel networks exhibited similar complexity, and this was reflected in many of the network topological characteristics. For example, the number of interactions (edges) and taxa was similar between networks (Table 2). Notably, the core community network exhibited a greater clustering coefficient and average number of neighbours. On the local (nodal) level, the core community exhibited greater neighbourhood connectivity (0.04 vs. 0.01, *p* < 0.05) and radiality (0.55 vs. 0.48, *p* < 0.001), although no significant differences were observed in the average shortest path lengths (Table 3). Both networks were dominated by a high proportion of positive interactions (95%) suggesting a potential instability in both network structures.

**TABLE 2:** Global network characteristics of apple microbial communities.

Network (global) topological characteristics	Core tissue communities	Peel tissue communities
Number of taxa	317	308
Number of interactions	367	344
Average number of neighbours	2.31	2.22
Network diameter	6	6
Characteristic path length	1.834	1.939
Clustering co‐efficient	0.016	0.006
Network density	0.004	0.004

**TABLE 3:** Node (local) topological characteristics of apple microbial community networks.

Node (local) topological characteristics	Core tissue communities	Peel tissue communities
Clustering coefficient*	0.04	0.01
Degree	2.31	2.22
Neighbourhood connectivity	3.48	3.25
Radiality***	0.55	0.48

*Note:* *, **, and *** indicate *p* < 0.05, *p* < 0.01, *p* < 0.001, respectively. Statistical significance was calculated via the Student *t*‐test.

To determine the taxa with the most significant influence on both networks, we examined taxa that were in the top 10% of interactions, closeness centralities, and betweenness centralities. In the core network, four such influential taxa were identified: *Izhakia, Aerococcus, Jatrophihabitans* and an unidentified genus of the *Sphingobacteriaceae* family. These did not overlap with the three influential taxa identified in the peel network, which were *Proteiniphilum*, an unidentified genus of the *Pasteurellaceae* family, and an unidentified member of the *Microbotryomycetes* class.

### 3.6 Functional Analysis of the Bacterial Communities of ‘Honeycrisp’ Tissues

**FIGURE 5:**
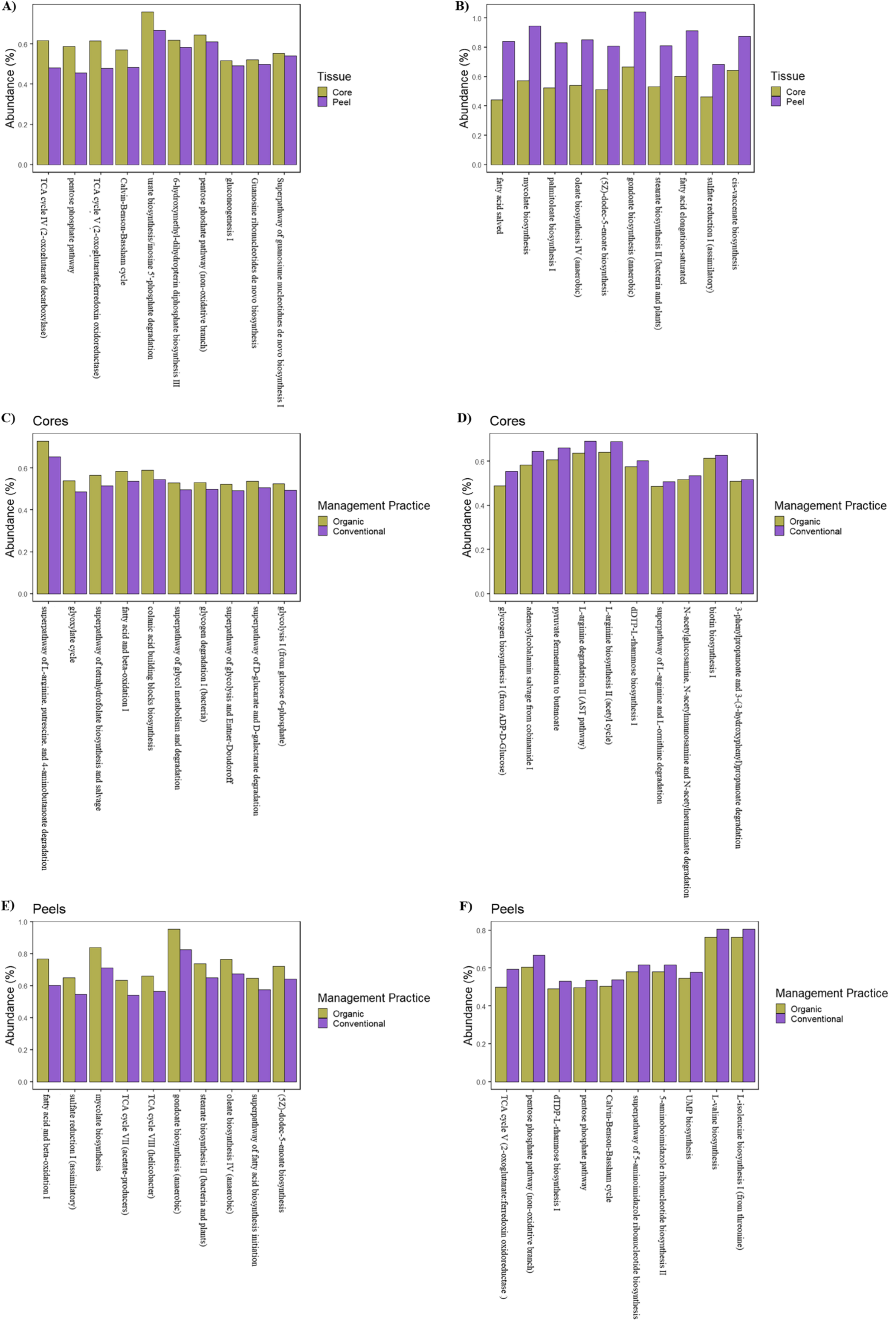
The 10 most abundant and significantly represented MetaCyc pathways predicted in the communities of core (A) and peel (B) tissues, organically (C) and conventionally (D) managed core tissues, and organically (E) and conventionally (F) managed peel tissues. Significant results were based on a Benjamini‐Hochberg multiple test correction of a Welch's *t*‐test (*p* < 0.05).

Significant differences were identified in the predicted metabolic pathways encoded in the metagenomes of apple core and peel tissues. Out of 396 individual metabolic pathways identified in the dataset, 315 were differentially represented between the two tissue types (we.eBH *p* < 0.05, Table S5A). The metabolic pathways with the greatest overrepresentation in the communities of core tissues were the TCA cycles IV and V, the oxidative and non‐oxidative branches of the pentose phosphate pathway, the Calvin‐Benson Bassham Cycle, urate biosynthesis/inosine 5′‐phosphate degradation, 6‐hydroxymethyl‐dihydropterin diphosphate biosynthesis III, gluconeogenesis I, guanosine ribonucleotides de novo biosynthesis and the superpathway of guanosine nucleotides de novo biosynthesis I, respectively (Figure 5A). In comparison, the metabolic pathways with the greatest overrepresentation in peel tissue communities were cis‐vaccenate biosynthesis, sulphate reduction I (assimilatory), fatty acid elongation (saturated), stearate biosynthesis II, gondoate biosynthesis (anaerobic), (5Z)‐dodec‐5‐enoate biosynthesis, oleate biosynthesis IV (anaerobic), palmitoleate biosynthesis I (from (5Z)‐dodec‐5‐enoate), mycolate biosynthesis and fatty acid salvage, respectively (Figure 5B).

Among the 385 metabolic pathways identified in the tissue communities of organically and conventionally managed cores, 171 pathways were differentially represented between the two management systems (we.eBH *p* < 0.05, Table S5B). In organically managed core tissues the 10 most overrepresented metabolic pathways included the superpathway of L‐arginine, putrescine, and 4‐aminobutanoate degradation glyoxylate cycle, the superpathway of tetrahydrofolate biosynthesis and salvage, fatty acid and beta‐oxidation I, colanic acid building blocks biosynthesis, the superpathway of glycol metabolism and degradation, glycogen degradation, the superpathway of glycolysis and Entner‐Doudoroff, the superpathway of D‐glucarate and D‐galactarate degradation, and glycolysis I (from glucose 6‐phosphate), respectively (Figure 5C). In comparison, in conventionally managed core tissue, the most enriched metabolic pathways were 3‐phenylpropanoate and 3‐(3‐hydroxyphenyl) propanoate degradation to 2‐oxopent‐4‐enoate, biotin biosynthesis I, the superpathway of N‐acetylglucosamine, N‐acetylmannosamine and N‐acetylneuraminate degradation, the superpathway of L‐arginine and L‐ornithine degradation, dTDP‐L‐rhamnose biosynthesis I, L‐arginine biosynthesis II (acetyl cycle), L‐arginine degradation II (AST pathway), pyruvate fermentation to butanoate, adenosylcobalamin salvage from cobinamide I and glycogen biosynthesis I (from ADP‐D‐Glucose), respectively (Figure 5D).

In the peel tissue communities of organically and conventionally managed fruit, a total of 384 metabolic pathways were identified, with 248 pathways being differentially represented between the two management systems (we.eBH *p* < 0.05, Table S5C). The metabolic pathways most enriched in organically managed peel tissue communities compared to conventionally managed were: fatty acid and beta‐oxidation I, sulphate reduction I (assimilatory), mycolate biosynthesis, TCA cycle VII and VIII, gondoate biosynthesis (anaerobic), stearate biosynthesis II (bacteria and plants), oleate biosynthesis IV (anaerobic), the superpathway of fatty acid biosynthesis initiation, and (5Z)‐dodec‐5‐enoate biosynthesis, respectively (Figure 5E). On the other hand, the metabolic pathways with the greatest overrepresentation in conventionally managed compared to organically managed peel tissue communities were L‐valine biosynthesis, L‐isoleucine biosynthesis I (from threonine), UMP biosynthesis, the superpathway of 5‐aminoimidazole ribonucleotide biosynthesis, 5‐aminoimidazole ribonucleotide biosynthesis II, Calvin‐Benson‐Bassham cycle, the oxidative and non‐oxidative branches of the pentose phosphate pathway, dTDP‐L‐rhamnose biosynthesis I, and TCA cycle V (2‐oxoglutarate:ferredoxin oxidoreductase), respectively (Figure 5F).

### 3.7 CORE Microbiome of ‘Honeycrisp’ Apples in Atlantic Canada

The CORE bacterial microbiome of the ‘Honeycrisp’ apple in this study consisted of 28 ASVs which were present in over 75% of samples across tissue types and abiotic factors. These ASVs in turn corresponded to 19 genera and two bacterial families (Table 4). In a previous study examining the fungal microbiome within the same tissue samples (McLaughlin et al. 2023), we observed that the CORE fungal microbiome of the ‘Honeycrisp’ apple comprised six fungal genera, though none of these genera appear to be influential taxa within either network. Thus, the CORE microbiome of ‘Honeycrisp’ apples in Maritime Canada comprises 25 identified genera, three unidentified members of two bacterial families, and an unidentified genus of the *Capnodiales* order.

**TABLE 4:** The Relative abundance of CORE bacterial ASVs and their corresponding genera of ‘Honeycrisp’ apple fruit tissue communities in the Atlantic Maritime Ecozone.

Relative abundance (%)	Genus
10.04	*Allorhizobium–Neorhizobium–Pararhizobium–Rhizobium*
9.22	*Pantoea*
6.80	*Curtobacterium*
6.52	*Sphingomonas*
5.94	*Pseudomonas*
3.74	*Brevundimonas*
2.58	*Pseudomonas*
2.19	*Sphingomonas*
1.37	*Sphingobium*
1.13	*Massilia*
1.11	Unidentified Genus of the Microbacteriaceae Family
1.10	*Massilia*
0.80	*Methylobacterium–Methylorubrum*
0.65	*Methylobacterium–Methylorubrum*
0.61	*Pedobacter*
0.60	1174‐901‐12 of the Beijerinckiaceae family
0.49	*Staphylococcus*
0.47	*Hymenobacter*
0.35	*Duganella*
0.35	*Aeromicrobium*
0.34	*Corynebacterium*
0.25	1174‐901‐12 of the Beijerinckiaceae family
0.24	Unidentified Genus of the Comamonadaceae Family
0.22	*Methylobacterium–Methylorubrum*
0.19	*Enhydrobacter*
0.15	P3OB‐42 of the Myxococcaceae Family
0.13	*Novosphingobium*
0.13	Unidentified Genus of the Comamonadaceae Family

## 4 Discussion

In this study, we expand upon our previous work (McLaughlin et al. 2023) to demonstrate tissue‐specific differences in the diversity and structure of bacterial communities of ‘Honeycrisp’ apple fruit and explore the relative impact of differing environmental conditions between growing seasons, geographical locations, and management practices on apple fruit bacterial communities. Furthermore, for the first time, we describe microbial relationships that define apple core and peel tissue community networks with the goal of providing a framework for understanding the dynamics that influence apple microbial communities.

For decades, growers have relied on chemical fungicides to achieve pre‐ and post‐harvest disease control in apple orchards. However, increasing concerns over the negative environmental and human health impacts of these products have led to stricter regulations on their use, highlighting the need for alternative approaches to disease control (Ragsdale and Sisler 1994; Steinhauer et al. 2018). In this respect, it has been suggested that the microbiome could be harnessed to control disease—either through maintaining the microbiome in a “healthy” state (Berg et al. 2020), or by actively tailoring the microbiome to resist disease (Berg et al. 2020). This approach is considered particularly promising for managing fungal post‐harvest diseases in fruit (Droby and Wisniewski 2018). However, in order to achieve this, a thorough understanding of how biotic and abiotic factors influence the microbiome of apple fruit will be necessary. Numerous studies have demonstrated variation in the diversity and/or structure of the apple fruit microbiome in response to biotic and abiotic factors, including genotype, tissue type, geographical location, management practices, seasonal environmental differences, pre‐storage treatments, storage duration, post‐harvest processing, and the growing system (organic vs. conventional) (Abdelfattah et al. 2016, 2020, 2021; Wassermann, Muller et al. 2019; Bosch et al. 2021; Britt et al. 2021; Wicaksono et al. 2022, 2023; McLaughlin et al. 2023, 2024). However, comparing the relative impact of these variables is difficult, as they are often not examined in the same study or sample set. In this study, we examined the impact of tissue type, seasonal environmental conditions, and organic versus conventional management practices on the diversity and structure of apple fruit microbial communities within a single experiment.

### 4.1 Core Fruit‐Associated Microbiota

A small number of microbiota can be consistently identified in specific plant tissue samples, and there is some evidence to suggest that these “core microbiota” may support and promote plant health (Berg et al. 2020). In a global analysis of the microbiome of the ‘Royal Gala’ apple, two such core bacteria were identified in at least 75% of all samples: *Sphingomonas* and *Methylobacterium* (Abdelfattah et al. 2021). In contrast, we identified 19 bacterial genera and 2 bacterial families as “core microbiota” in ‘Honeycrisp’ apple, which included both *Sphingomonas* and *Methylobacterium*. There are several factors that could explain the larger number of core microbiota in this study. The global analysis previously demonstrated that the geographical effect on the variation of apple microbial communities is positively correlated with the distance. Therefore, less variation would be expected in the present study due to its comparatively restricted geographical range. Furthermore, genotype‐specific influences on apple fruit microbiota have previously been described, and ‘Honeycrisp’ has been demonstrated to be genetically distinct from many of the most popular cultivars in the United States Department of Agriculture's collection, including ‘Gala’ (Liu et al. 2018; Bosch et al. 2021; Britt et al. 2021; Migicovsky et al. 2021). Given the significant influences of genotype on overall apple fruit microbiota, it is possible that some cultivars may retain unique “core microbiota”. Nevertheless, as we observed with some fungal genera in our previous study (McLaughlin et al. 2023), it is possible that some core bacterial genera may be conserved across cultivars, and a broader study encompassing many distantly related cultivars may be required to determine the “core microbiota” conserved across genotypes.

### 4.2 Tissue‐Specific Variation

A number of studies have shown tissue‐specific differences in apple fruit fungal and/or bacterial community composition (Wassermann, Muller et al. 2019; Abdelfattah et al. 2021; McLaughlin et al. 2023, 2024). This has led to the suggestion that spatial differences in nutrient availability, morphology, and microhabitat may promote or prevent the colonization and growth of individual microbiota in a tissue‐specific manner and thus may significantly influence microbial community composition. In this study, we provide further evidence for tissue‐specific differences in bacterial community structure and diversity. Individually, variations between tissue types accounted for approximately 32% of the differences in bacterial communities and were the most significant factor influencing bacterial community variation in this study. This was greater in fungal communities, in which tissue type accounted for only 7% of the differences in the data set (McLaughlin et al. 2023). Few studies have previously compared the influence of tissue‐specific differences on both fungal and bacterial community composition, although in a global study of the microbiome of ‘Gala’ fruit, Abdelfattah et al. (2021) observed that tissue type could account for approximately 3% and 5% of the variations in fungal and bacterial communities, respectively. Additionally, Shannon diversity was not influenced by tissue type unless the data were examined at the orchard level, in stark contrast to bacterial Shannon diversity which was significantly influenced by tissue type regardless of sampling location (Abdelfattah et al. 2021). Our study demonstrated key differences in bacterial community diversities between core and peel tissues, with core tissues being defined by a lower number of ASVs, evenness, as well as Shannon diversity, whereas these indices were not significantly different in the fungal communities of these core and peel tissues (McLaughlin et al. 2023). Taken together, it seems apparent that apple bacterial communities may be more susceptible to the influence of unique tissue micro‐environments within the fruit when compared with their fungal counterparts. Given the significance of tissue type in determining the diversity and structure of the bacterial microbiome of apple fruit, it is apparent that future studies should include several tissue types, as single tissue analyses will not accurately reflect the dynamics of the bacterial communities within the fruit.

### 4.3 Effects of Environmental Conditions Between Growing Seasons

Despite a significant decrease in precipitation in 2020 compared with 2019, the environmental conditions of the growing season explained only a small proportion of the variation in the bacterial communities of either tissue type, especially when compared with the stronger effects seen in the fruits‐associated fungal communities (McLaughlin et al. 2023). Several studies have demonstrated higher relative stability of the bacterial communities of apple tissues compared with their corresponding fungal communities under different storage conditions and pre‐storage treatments (Wassermann, Kusstatscher et al. 2019; Abdelfattah et al. 2020), as well as geographical locations (Abdelfattah et al. 2021). In this study, the effect of the environmental conditions of the growing season on the variation of bacterial communities was only significant in peel tissues. In addition, the communities of peels sampled in 2020 exhibited significantly lower Shannon diversity, evenness, and observed features when compared with those sampled in 2019. Changes in the environmental conditions of the growing season have previously been demonstrated to significantly influence the structure and diversity of apple fruit peel bacterial communities (Bosch et al. 2021; Britt et al. 2021). Differential exposure to the changing environments could partially explain the relative stability of the core bacterial communities across the growing season compared with those of peel tissue, with the stem and calyx end of the apple providing a more sheltered microhabitat, while peel tissue would be exposed more to these environmental elements. The relative stability of bacterial communities of core tissues observed between growing seasons in this study may be a useful trait to harness for biological control of post‐harvest disease, as the calyx and stem ends of apple fruit have been observed to be particularly vulnerable to the development of post‐harvest rot in storage.

### 4.4 Management Practices

This study aimed to assess the influence of organic and conventional management practices on the bacterial communities of ‘Honeycrisp’ apple fruit at harvest. Previous research has shown that organically managed fruit often harbour bacterial communities with higher alpha diversity compared to those from conventionally managed fruits. In ‘Arlet’ apples following preparation for sale, all organic tissues (except the calyx end tissue) exhibited significantly greater Shannon diversity than their conventional counterparts. Likewise, in the peel tissues of ‘Ariane’, ‘Otava’ and ‘Topaz’, organic management was also associated with significantly higher Shannon diversity (Wassermann, Muller et al. 2019; Bosch et al. 2021; Britt et al. 2021). Although management practices had a clear and significant impact on bacterial communities of both core and peel tissues in the present study, their effects on diversity were not consistent between tissue types. Specifically, conventionally managed peel tissues exhibited significantly greater diversity than their organically managed counterparts, whereas conventionally managed core tissues were less diverse than organically managed cores. Thus, the diversity of bacterial communities in apple fruit may not always positively correspond to organic practices. A possible explanation for the increased alpha diversity observed in conventionally managed peel tissues in this study could be a combination of increased ease of colonization due to a loss of competition in peel tissue surfaces, and the greater exposure of peel tissues to the external environment. Antibiotics such as *Streptomycin* are typically applied during the flowering stage in conventional orchards, providing ample opportunity for subsequent bacterial colonization of the apple fruit throughout the growing season. Nevertheless, this study provides further evidence that management practices significantly influence the structure and diversity of the bacterial communities of apple fruit.

### 4.5 Network Characteristics

Microbial communities are shaped by networks of co‐operative and competitive interactions among individual taxa, which can strongly influence their function, stability and population dynamics. Consequently, a shift in the relative abundance of even a single microbe can trigger cascading effects on other members of the community (Czárán et al. 2002; Kinkel et al. 2011; Friedman et al. 2017; Banerjee et al. 2018). The stability of the microbial communities can be predicted by factors such as the ratio of cooperative and competitive interactions, as well as the connectance and modularity of the microbial community (Coyte et al. 2015; Herren and McMahon 2017; de Vries et al. 2018; Hernandez et al. 2021). Typically, a lower proportion of positive interactions, greater modularity, and increased competition are associated with improved network stability. Though positive interactions may promote network functionality (functional redundancy), a high degree of positive interactions may indicate the establishment of co‐dependency and associated positive feedback loops between taxa within the network. In such cases, the loss in abundance of a single taxon may disproportionally affect others, and lead to network instability (Coyte et al. 2015). Conversely, although negative interactions are important for the stability of microbial networks, a high proportion of negative interactions can lead to a ‘negative complementary effect’ and cause ecosystem collapse (Becker et al. 2012). Therefore, the stability of microbial networks may depend on the presence of both positive and negative interactions, and the dominance of either may lead to microbial network instability. A loss of network stability within the fruit microbiome may in turn lead to the development of an imbalance in the microbial community known as dysbiosis which has been correlated with increased susceptibility to disease. For instance, a reduction in the relative abundance of the Gram‐positive bacteria, Firmicutes and Actinobacteria phyla in the rhizosphere was associated with the development of bacterial wilt of tomato, caused by *Ralstonia solanacearum* , despite the fact that there were no significant differences in the abundance of this pathogen between diseased and healthy tomato plants (Lee et al. 2021). Likewise, post‐harvest stem end rot in mango fruit, caused by *A. alternata* , has been positively correlated with a loss in microbial diversity (Diskin et al. 2017). Therefore, maintaining the stability of apple fruit microbial networks may prove beneficial in disease reduction strategies. In this study, we sought to describe the microbial ecological networks in apple core and peels and determine the relative stability of these networks.

Both core and peel networks exhibited a high degree of positive interactions, suggesting a high degree of co‐dependency and low network stability. As with bacterial community diversity and variability, it is clear that the tissue microenvironment significantly influences community network dynamics, with core community networks exhibiting higher complexity. There was no overlap in the most influential taxa in the peel and core community networks. These tissue‐specific differences in key influential taxa and network complexity may indicate additional challenges for future aspirations of post‐harvest disease control through maintenance or manipulation of the apple fruit microbiome.

### 4.6 Functional Characteristics of Apple Fruit Communities

The tissue micro‐environment strongly shaped the structure and diversity of apple‐associated microbial communities, functional analysis of the bacterial metagenomes from ‘Honeycrisp’ core and peel tissues revealed that more than 64% of the predicted functional pathways were differentially represented between these niches. In core tissue communities, pathways related to sugar production as well as ATP and NADH generation were overrepresented compared to those in peel tissues. Therefore, the bacterial communities of the core tissue environment appear to be characterized by greater energy production, carbon fixation and biosynthesis compared to the communities of peel tissues. The overrepresentation of these pathways in the communities of core tissues could be driven by greater nutrient and starch availability compared to the peel micro‐environment. Previous studies have shown increasing nutrient gradients from the core to the peel tissues and from the stem to the calyx end of the fruit (Neilsen et al. 2003; Doerflinger et al. 2015). In contrast, many of the most abundant functional pathways overrepresented in the communities of peel tissues were associated with the integrity, structure and stability of bacterial membranes. These pathways included the synthesis of cis‐vaccenate, palmitoleate and fatty acid elongation and salvage. Cis‐vaccenate has been associated with membrane fluidity and adaptation to changing temperatures in *Escherichia coli* (Hoogerland et al. 2023). Likewise, the presence of the fatty acid palmitoleate has been associated with improved *E. coli* membrane fluidity at low temperatures, while *E. coli* mutants unable to synthesize palmitoleate demonstrate increased susceptibility to antibiotics (Vorachek‐Warren et al. 2002). Consequently, the peel microbial communities may be composed of taxa better adapted to a rapidly changing and potentially stressful external environment.

Organic and conventional management practices significantly influence the functional metabolic pathways of the apple fruit microbiome, with 44% and 64% of predicted functional pathways being differentially represented between these management systems in core and peel tissue communities, respectively. One key aspect in which conventionally and organically managed core microbial communities differ is their utilization of glycogen, which serves as an energy reserve in bacteria (Strange 1968). In the communities of organically managed core tissues, the degradation of glycogen appears to be promoted. Conversely, glycogen biosynthesis appears to be favoured in the communities of conventionally managed core tissues. This suggests relatively high energy requirements, or a lack of available nutrients, in organically managed core tissues, possibly as a result of increased competition in these communities. The same pattern was not apparent in the communities of organically and conventionally managed peel tissues. In this respect, the communities of organically managed peels were associated with increased biosynthesis of fatty acids and fatty acid oxidation, suggesting a greater emphasis on membrane integrity and utilisation of environmental fatty acids to produce energy in the organic peel micro‐environment (Yao and Rock 2015; Jimenez‐Diaz et al. 2017). In contrast, many of the most significant metabolic pathways which are relatively higher in the communities of conventionally managed peel tissues involve the production of sugars and energy (including both branches of the pentose phosphate pathway, the TCA cycle and the Calvin‐Benson‐Bassham Cycle) as well as the production of proteins (L‐valine biosynthesis, L‐isoleucine biosynthesis I (from threonine)) and ribonucleotides (UMP biosynthesis, 5‐aminoimidazole ribonucleotide biosynthesis), suggesting that the community is geared towards growth and metabolism. Nevertheless, management practices significantly alter the metabolic functions of apple microbial communities, though the impact of these management practices appears to differ between specific fruit tissue micro‐environments.

In summary, the findings of this study show that different orchard management strategies and environmental conditions shape bacterial communities associated with apple fruit in a tissue‐specific way, influencing both microbiome composition and functional potential. This emphasizes the complexity of plant–microbe interactions as well as the significance of taking into account diverse tissue types when studying fruit microbiomes. These findings have practical implications for promoting, beneficial microbial communities and improving postharvest fruit disease management. More broadly, this study provides a framework for understanding how different management and environmental factors influence crop‐associated microbiomes, with implications for disease management, fruit quality, and long‐term production across a variety of horticultural systems.

## 5 Conclusions

Maintenance or manipulation of the fruit microbiome is viewed as a new frontier in eco‐friendly post‐harvest disease management. Our work reveals significant alterations in the composition of bacterial communities of ‘Honeycrisp’ apple fruit and demonstrates that environmental and management factors influence these communities in a manner dependent on the tissue micro‐environment. Differences between core and peel tissue communities were reflected in their microbial networks, and predicted functions. Taken together, these findings underscore the significant challenges involved in harnessing the fruit microbiome for disease control, particularly in the pre‐harvest context. Microbiome manipulation for the purposes of disease management may be better suited to post‐harvest applications where external variables can be more easily controlled and distinct micro‐environments—namely the calyx and stem ends of apple fruit—are known to be especially vulnerable. To effectively utilize the apple fruit microbiome for disease prevention in this context, it will be essential to account for tissue‐specific variations in microbial diversity, structure, and function and determine the relationship of these factors with susceptibility to post‐harvest diseases.

## Author Contributions

Michael Shayne McLaughlin: Formal analysis (equal); investigation (equal); methodology (equal); writing – original draft (equal); writing – review and editing (equal). Svetlana N. Yurgel: Formal analysis (equal); validation (equal); writing – review and editing (equal). Pervaiz A. Abbasi: Project administration (equal); supervision (equal); Shawkat Ali: Conceptualization (equal); formal analysis (equal); investigation (equal); methodology (equal); supervision (equal); validation (equal); writing – review and editing (equal); Project administration (equal).

## Conflicts of Interest

The authors declare no conflicts of interest.

## Supporting information


**TABLE S1:** Relative abundance of resolved taxa within the dataset.
**TABLE S2:** The 51 differentially represented bacterial genera between core and peel tissues as generated by Aldex2.
**TABLE S3A:** The 12 differentially represented bacterial genera between management practices in core tissues by Aldex2.
**TABLE S3B:** The 12 differentially represented bacterial genera between management practices in peel tissues by Aldex2.
**TABLE S4A:** The two differentially represented bacterial genera between growing seasons in core tissues by Aldex2.
**TABLE S4B:** The 21 differentially represented bacterial genera between growing seasons in peel tissues by Aldex2.
**TABLE S5A:** The differentially represented predicted metabolic pathways between core and peel tissues by Aldex2.
**TABLE S5B:** The differentially represented predicted metabolic pathways between organically and conventionally managed core tissues by Aldex2.

## Data Availability

The data that support the findings of this study are openly available in NCBI at https://www.ncbi.nlm.nih.gov/sra/PRJNA1213154, reference number PRJNA1213154.
